# Comparing the Effects of Symbiotic Algae (*Symbiodinium*) Clades C1 and D on Early Growth Stages of *Acropora tenuis*

**DOI:** 10.1371/journal.pone.0098999

**Published:** 2014-06-10

**Authors:** Ikuko Yuyama, Tomihiko Higuchi

**Affiliations:** Graduate School of Science and Technology, Shizuoka University, Shizuoka, Japan; Pennsylvania State University, United States of America

## Abstract

Reef-building corals switch endosymbiotic algae of the genus *Symbiodinium* during their early growth stages and during bleaching events. Clade C *Symbiodinium* algae are dominant in corals, although other clades — including A and D — have also been commonly detected in juvenile Acroporid corals. Previous studies have been reported that only molecular data of *Symbiodinium* clade were identified within field corals. In this study, we inoculated aposymbiotic juvenile polyps with cultures of clades C1 and D *Symbiodinium* algae, and investigated the different effect of these two clades of *Symbiodinium* on juvenile polyps. Our results showed that clade C1 algae did not grow, while clade D algae grew rapidly during the first 2 months after inoculation. Polyps associated with clade C1 algae exhibited bright green fluorescence across the body and tentacles after inoculation. The growth rate of polyp skeletons was lower in polyps associated with clade C1 algae than those associated with clade D algae. On the other hand, antioxidant activity (catalase) of corals was not significantly different between corals with clade C1 and clade D algae. Our results suggested that clade D *Symbiodinium* algae easily form symbiotic relationships with corals and that these algae could contribute to coral growth in early symbiosis stages.

## Introduction

Mass bleaching of corals caused by global warming threatens the degradation of reef ecosystems worldwide [1]. Coral bleaching involves a breakdown of the symbiotic relationships between reef-building corals and their symbiotic algae, dinoflagellates such as *Symbiodinium*. The genus *Symbiodinium* is currently classified into nine clades (A–I) [2]–[4]. Clade C is most often associated with corals, although corals occasionally switch their symbiotic algae. Especially after bleaching events, clade D *Symbiodinium* has been detected in corals [5]–[6]. Flexibility in symbiotic associations has also been observed in the early growth stage of Acroporid corals infected by *Symbiodinium* algae from the environment (horizontal transmission). Some studies have shown that juvenile Acroporid corals were first dominated by nonhomologous adult *Symbiodinium* algae from clade A or D, and later by clade C algae, which had an adult homologous association [7]–[9]. Conversely, Little (2004) and Littman et al. (2010) found that Acroporid juvenile polyps, around one- month old, were able to acquire clade C *Symbiodinium* algae [10]–[11]. Thus, corals can change their dominant symbiotic algae depending on their environment and growth stage. However, these previous studies have only shown molecular data of *Symbiodinium* clade within field corals. There were no studies comparing the increased rate of each *Symbiodinium* clade in juvenile polyps.

The physiological properties of corals may be influenced by their dominant clade of endosymbiotic algae. Some studies have shown that clade D *Symbiodinium* algae are thermally tolerant and increase coral resistance to elevated sea surface temperatures [6], [12]. Baker et al. (2004) showed that in 1997, corals containing clade D *Symbiodinium* algae were unaffected by bleaching, while corals associated with clade C algae were severely bleached [5]. Adult *Acropora millepora* corals have shown an increase in thermal tolerance, by 1–1.5°C, after changing their dominant symbiont algae from clade C to clade D [13]. On the other hand, it has been reported that juvenile *Acropora tenuis* polyps hosting clade C1 algae had greater thermal tolerances than those associated with clade D algae [14]. Genotypic differences of symbiotic algae could also influence the growth rates of corals. Acroporid corals with clade C *Symbiodinium* algae showed a higher growth rate than those associated with clade D algae [15], [10]. In addition, fluorescent protein in juvenile polyps was also changed the amount by endosymbiotic *Symbiodinium* clade [16]. Yuyama et al., (2012) showed that the expression pattern of fluorescent protein homolog and some stress responsive genes were different between clade A and clade D symbiosis [16].

In the present study, we exposed aposymbiotic juvenile polyps to monoclonal cultures of *Symbiodinium* clade C1 and clade D, and we used these polyps as model symbiosis system. Previous laboratory experiments demonstrated that immediately after metamorphosis, juvenile *Acropora tenuis* polyps could form symbiotic relationships with *Symbiodinium* algae in clades A and D, but not with those in clade C [16]–[17]. Thus, we conducted a long-term laboratory experiment for cultivating corals associated with clade C1 algae. We compared the growth rate of the skeleton and fluorescence of polyps between corals associated with algae in clades C1 and D. In addition, photosynthetic activity of symbiont could produce reactive oxygen species that might affect the coral condition [18]. For further understanding the different effect of each symbiont clade on corals, we also investigated the antioxidant activity.

## Materials and Methods

### Animals and algae

Colonies of *A. tenuis* were collected at the Akajima Marine Science Laboratory (Okinawa, Japan) [with permission from the Okinawa Prefectural Government]. Colonies spawned on 4th June 2010. Collection of *A. tenuis* larvae was performed as previously described [19]. Seven days after spawning, larvae were exposed to 2 µM Hym 248 to induce metamorphosis in containers 55 mm in diameter [18]. Filtered (pore size: 0.22 µm) seawater was used to exclude *Symbiodinium* algae prior to introduction of specific clades. In this study, two cultured *Symbiodinium* strains in clade C1 and clade D were used as some studies reported that *A. tenuis* were capable of establishing symbiosis with these clades in natural conditions [14]. The *Symbiodinium* strains CCMP2466 (clade C1) and CCMP2556 (clade D) were obtained from the Bigelow Laboratory for Ocean Sciences (West Boothbay Harbor, ME, USA; https://ccmp.bigelow.org/) and cultured in IMK medium (Wako Chemicals, Osaka, Japan) and antibiotics (kanamycin 20 µg/mL and ampicillin 50 µg/mL) at 24°C under a 12-h light (20 µE/m^2^/s): 12-h dark cycle. Juvenile polyps were cultured in Petri dishes containing filtered (pore size: 0.22 µm) seawater at 24°C under a 12-h light (50 µE/m^2^/s): 12-h dark cycle. Cells of each strain of *Symbiodinium* algae (approximately 1,000 cells per polyp) were introduced to *A. tenuis* primary polyps 10 days after metamorphosis. Each *Symbiodinium* culture was subsequently introduced to Petri dishes containing polyps every day. Approximately 10–20 larvae have settled in each dish. A portion of the juvenile polyps were maintained in an aposymbiotic state and was used for experiments. Six dishes were used each treatment (inoculation with clade C1, inoculation with clade D, aposymbiotic), and seawater was changed on a daily basis. Each container was covered with plastic wrap to prevent cross-contamination between clades.

### Microscopic observation

Juvenile polyps were observed with a stereomicroscope during the incubation period. Color micrographs of several polyps were taken with a digital scanning microscope (model VHX-1000; Keyence, Tokyo, Japan) to evaluate the density of symbionts. Micrographs of polyps were taken 20, 58, 80, and 120 days after inoculation with *Symbiodinium* algal cultures. Epifluorescence photomicrographs of polyps were taken using a Multizoom AZ100 microscope (Nikon, Tokyo, Japan) using a digital camera (Digital Slight DA-L1; Nikon) 20, 58, and 80 days after inoculation. Approximately 10 polyps were photographed for each treatment.

### *Symbiodinium* cell count

Polyps were fixed in 3% formaldehyde 20, 58, 80, and 120 days after inoculation with *Symbiodinium*. Subsequently, samples were decalcified using the decalcification solution, 0.5 M ethylenediaminetetraacetic acid (EDTA) for 2 days. Each polyp was placed in a 1.5-mL tube containing 0.01% TritonX, and homogenized. Algal cells in homogenate were counted using a hemocytometer (Thomas Scientific, Swedesboro, NJ). Each day, three or four polyps were used for counting *Symbiodinium* algal cells. Polyps associated with clade C1 algae had a low number of *Symbiodinium* cells in 20 days. Hence, the polyps were crushed using a cover glass onto a glass slide for counting endosymbiotic clade C1 cells in 20 days.

### Confirmation of endosymbiotic *Symbiodinium* clades

To confirm endosymbiotic algal clades, restriction fragment length polymorphism (RFLP) was performed. We used symbiotic polyps of 1- and 4-months old after inoculation with *Symbiodinium*. Three polyps were homogenized and DNA was extracted using a Plant Mini Kit (Qiagen, Valencia, CA). The SSU rDNA was amplified using the primers ss5z and ss3z according to Rowan and Powers (1991) [20]. For RFLP analysis of the SSU rDNA, 10 µL of product from each PCR amplification was digested with 1 µL *Taq*I enzyme (Takara, Ohtstu, Japan) for 3 h at 65°C in 10 µL of distilled H_2_O and 2 µL enzyme buffer solution. Reaction fragments were separated by electrophoresis and stained with ethidium bromide.

### Weight of coral skeleton

To estimate coral growth rates, skeletons were weighed using symbiotic polyps that had been stored for 105 and 135 days post-inoculation with *Symbiodinium* cultures. Aposymbiotic polyps that had been kept for 10 days after metamorphosis were also used (0 days after inoculation). We chosen 105 and 135 days, so as almost similar number of clades C and D *Symbiodinium* inside the polyp could be reached, to keep homogeneity for microscopic analysis and one month later the growth rate was assessed. In this year, we could not keep a lot of aposymbiotic polyps for long time, hence aposymbiotic polyps could not be used in this analysis. Corals were placed in 10% HCL and kept overnight to remove soft tissues, and dried for several days. Each coral skeleton was weighed with a supermicro scale (Sartorius AG, Göttingen, Deutschland).

### Catalase activity of corals

To investigate the level of oxidative stress inside the corals, the activity of the antioxidant enzyme, catalase, was measured using symbiotic polyps of 1- and 5-months post-inoculation. We measured catalase activity when fluorescent intensity of clade C1-polyps was highest (1 month post-inoculation), and when it became lower (5-months post-inoculation). Additionally, aposymbiotic polyps that had been incubated for the same period as 1 month-symbiotic polyps were also used. Catalase assays were conducted according to Higuchi et al. (2009) [21]. Two or three polyps were put in 0.2 mL of 100 mmol L^−1^ phosphate buffer (pH = 7.0) with 10 g L^−1^ of NaCl, containing 1% protease inhibitor cocktail (Sigma cat. No. P8340), and were then homogenized. The homogenates were further centrifuged twice at 600×*g* for 10 min to separate the supernatant and pellets. The supernatant was used to analyze protein and catalase activities of the host coral. Catalase activity was measured by the depletion of H_2_O_2_ at 240 nm [22]. All assays were conducted at 25°C, and enzyme activity was expressed as units (U) per mg protein. Protein content was determined by the Bradford assay [23]. The experiments were performed in triplicates.

### Statistical analysis

Statistical analyses were performed using JMP 8.0 (SAS institute, USA). We used two-way analyses of variance (ANOVA) with time and clade as explanatory variables and growth rates and catalase activity as dependent variables. The two-way repeated measures ANOVA with time and clade were used for *Symbiodinium* cell numbers. Student's t-test and post hoc Tukey–Kramer honestly significant difference (HSD) tests were used to determine clade differences.

## Results

### Increased rate of *Symbiodinium* in juvenile corals

**Figure 1:**
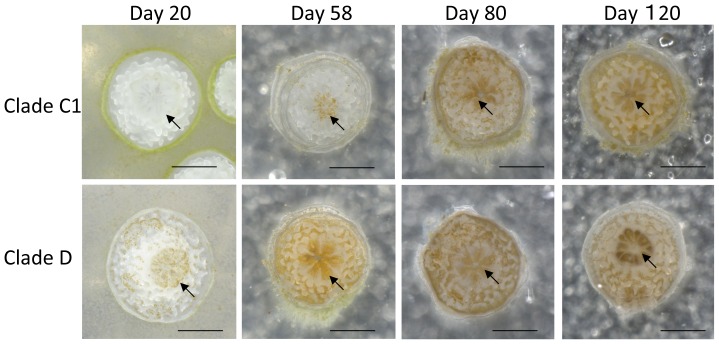
Juvenile polyps of *Acropora tenuis* colonized by clade C1 or clade D *Symbiodinium* monoclonal cells. Photographs of polyps were taken 20, 58, 80, and 120 days after inoculation. On day 20, polyps had only a few clade C1 cells but many clade D cells in their tentacles and body walls. The arrow indicates tentacles. Scale bar = 0.5 mm.

**Figure 2:**
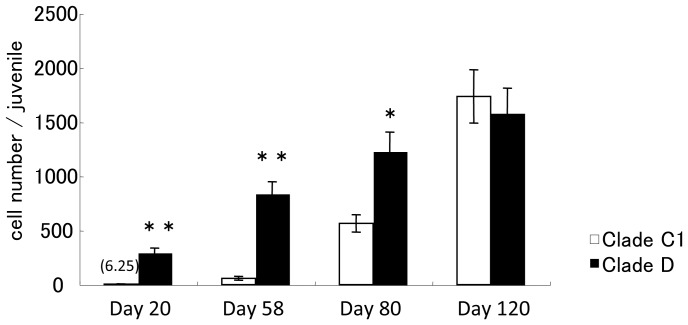
Number of symbiotic algal cells within clades C1 or D in juvenile polyps during the first 4 months after inoculation. Four juveniles were used to enumerate endosymbiont cells in each treatment and period. Values are means ± S.E. of the number of *Symbiodinium* algal cells per juvenile. The number above the bars for clade C1 at the day 20 indicates actual cell number. Asterisks indicate statistically significant differences resulting from t-tests: * *P*<0.05, ** *P*<0.01.

*Symbiodinium* strains in clades C1 and D were introduced to cultured *A. tenuis* polyps 10 days after metamorphosis, and the polyps were observed for 4 months. After 2 and 4 months, polyps were fixed and the symbiont clade was confirmed using Restriction Fragment Length Polymorphism (RFLP). The results of RFLP indicated that each polyp had mainly either clade C or D algal cells (Figure S1). Clade D cells existed in tentacles and thecal epithelia 2 days after inoculation, after which the number of symbiont cells in the polyps was increased. Numbers of *Symbiodinium* cells were significantly different between clades and time periods (repeated measures ANOVA; *P* = 0.0069 and *P* = 0.0002, respectively). Moreover, we found a significant synergistic effect of clade and time on *Symbiodinium* cell numbers (repeated measures ANOVA; *P* = 0.0020). On day 20, clade D cells had spread throughout the polyps (Fig. 1) and the number of clade D cells in the polyps was 293.66±42.02 (mean ± SE, *n* = 4; Fig. 2). However, only sporadic infection of clade C1 cells was observed in the tentacles (Fig. 1) on day 20 and polyps contained only 6.25±3.03 (*n* = 4) clade C1 cells. By day 58, polyps still contained only low numbers of clade C1 cells. Approximately 3 months after inoculation, the number of clade C1 cells had increased to half the number level of clade D cells in the polyps. After 4 months, the number of clade C1 cells in the polyps had increased to 1734.25±79.77 (*n* = 4) cells (Figs. 1 and 2). The number of clade D cells was significantly higher than that of clade C1 cells until 3 months post-inoculation (t-test; *P*<0.05). However, there was no significant difference between clades at 4 months. We further attempted to conduct inoculation experiments using 2-month-old aposymbiotic polyps. The results were similar to those of the experiment using 10-day -old aposymbiotic polyps, as described above (Figure S2).

### Green fluorescence

**Figure 3:**
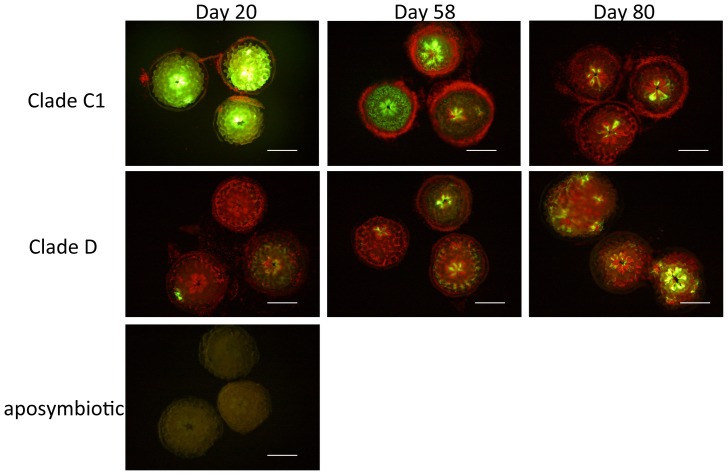
Fluorescence microscopic images of *Acropora tenuis* polyps associated with clade C1 or D *Symbiodinium* algae, and aposymbiotic polyps 20, 58, and 80 days after inoculation. Bright green fluorescence can be seen in the body walls and tentacles of polyps associated with clade C1 algae. On day 20, polyps associated with clade D algae show little green fluorescence, and aposymbiotic polyps have a pale fluorescence. Red fluorescence dots indicate the chlorophyll fluorescence of *Symbiodinium* algae. Polyps with clade C1 algae contain few *Symbiodinium* cells, although many cells are aggregated around the polyps on days 20 and 58 (Fig. 3), thought to be *Symbiodinium* algae released from the corals. Scale bar = 0.5 mm.

We observed green fluorescence in polyps associated with clades C1 and D (Fig. 3). Polyps with clade C1 algae exhibited bright green fluorescence throughout the whole body on day 20, which decreased gradually by days 58 and 80. The fluorescence of polyps associated with clade D algae existed partially during the experimental period, whereas aposymbiotic polyps displayed a watery green fluorescence throughout the body wall.

### Skeletal growth rate

**Figure 4:**
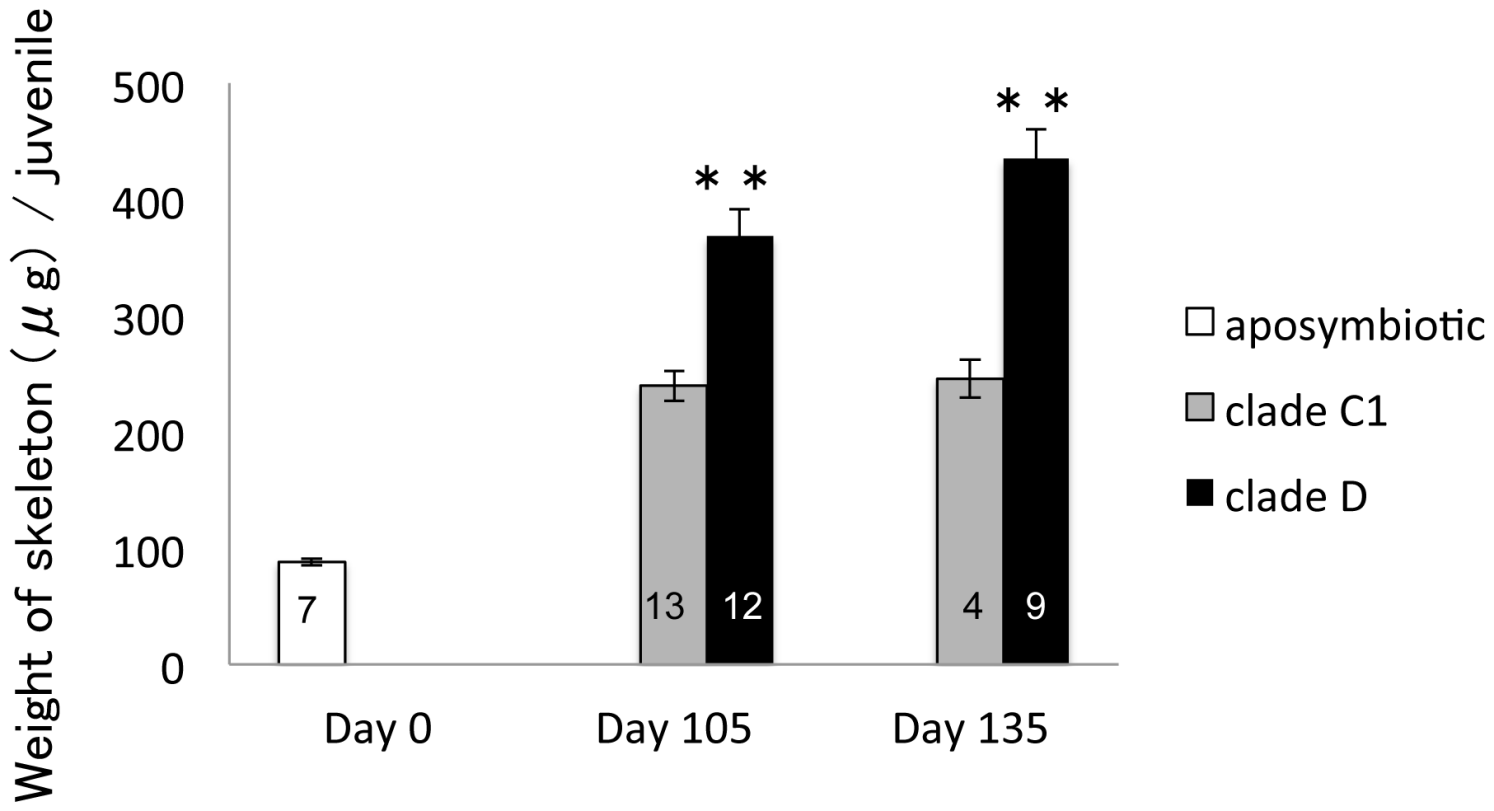
Skeleton weights (µg) of aposymbiotic and symbiotic polyps associated with clades C1 or D algae, 0, 105, and 135 days after inoculation. The number of polyps in each treatment are described in each bar graph. Values are means ± S.E. Asterisks indicate statistically significant differences resulting from t-tests: * *P*<0.05, ** *P*<0.01.

We compared the growth rate of polyps by measuring the weight of their skeletons (Fig. 4). The weight of aposymbiotic polyps 10 days after metamorphosis (0 day after inoculation with *Symbiodinium*) was 88.11±2.96 µg (*n* = 7). Juveniles with clade C1 or clade D algae were weighed 105 and 135 days after *Symbiodinium* inoculation. Between days 105 and 135, polyps with clade D algae were heavier than those with clade C1 algae (t-test; *P*<0.01); the weights of polyps with clade C1 algae remained largely consistent during this period. Polyps associated with clade C1 algae had growth rates of 6.3 µg/month and those associated with clade D algae had growth rates of 66.7 µg/month between 105 and 135 days.

### Catalase activity of corals

**Figure 5:**
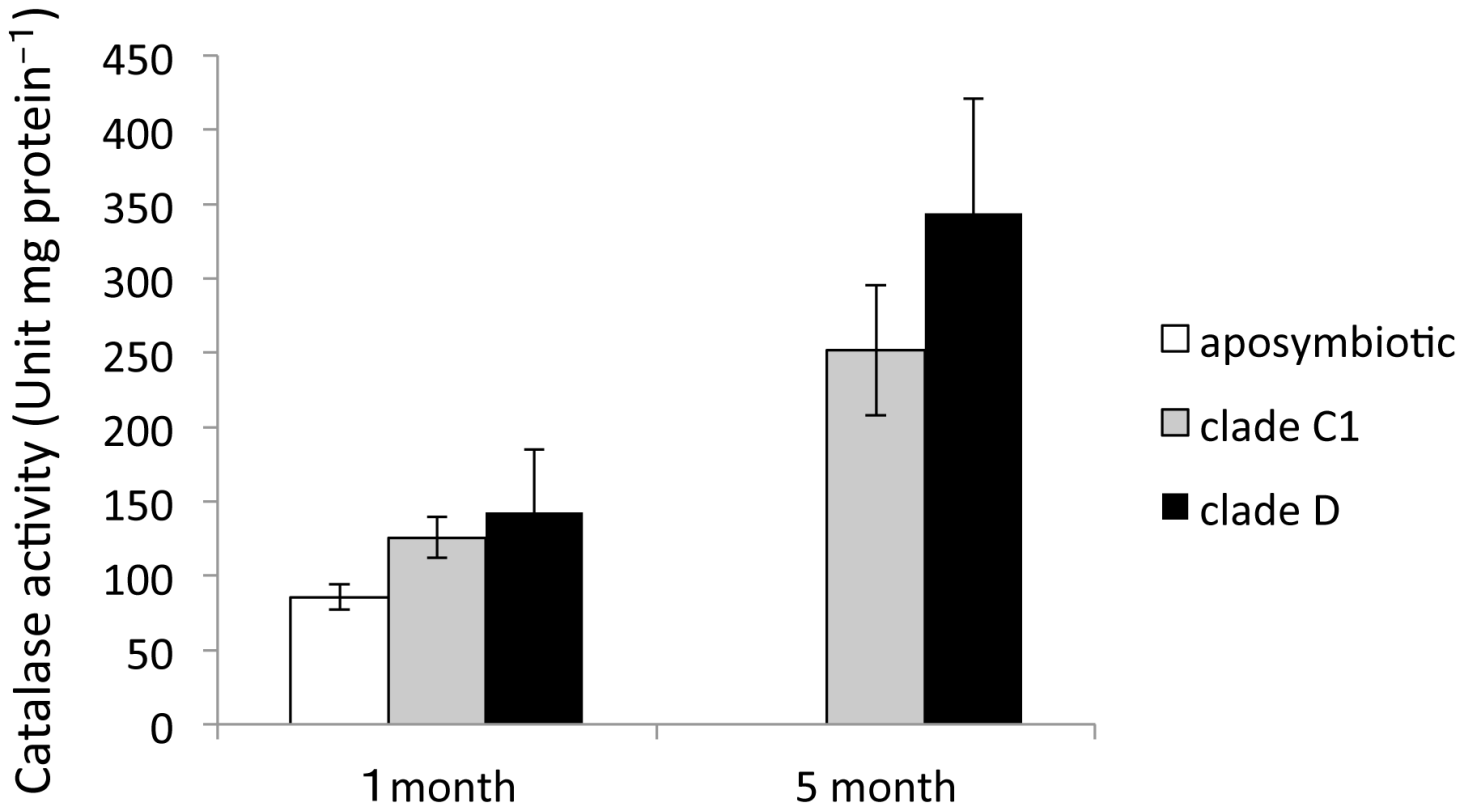
Catalase activity of aposymbiotic and symbiotic polyps associated with clade C1 or D algae, 1 and 5 months after inoculation. Each value represents the mean ± S.E. (*n* = 3). There is no significant differences between the treatments (Tukey–Kramer honestly significant difference (HSD) tests: *P*>0.05).

Catalase activity was investigated in aposymbiotic and symbiotic polyps (Fig. 5). One month after *Symbiodinium* inoculation, catalase activity was high in polyps with clade D algae (142.39±42.79, *n* = 3), and low in aposymbiotic polyps (85.53±8.62, *n* = 3). There was an increased activity 5 months after inoculation compared to 1 month after inoculation. Finally, polyps with clade D algae showed a higher level of activity (344.08±76.59, *n* = 3) than polyps with clade C1 algae (251.5±43.86, *n* = 3) at 5 months although there was no statistically significant difference between clades. Catalase activity increased significantly with incubation time (ANOVA, *P* = 0.011).

## Discussion

Past studies have shown that corals could acquire increased thermal tolerance by shifting their dominant symbiont algae clade C to clade D [13]. In the present study, we prepared the model system for coral–zooxanthellae symbiosis by infecting *A. tenuis* juveniles with monoclonal *Symbiodinium* culture in clade C1 or D. We discussed here the different effects of symbiotic algae clades C1 and D on physiological properties of juvenile polyps.

### Clades C1 and D *Symbiodinium* algae grew at different rates in coral polyps

Clade C1 *Symbiodinium* algae increased slowly in juvenile polyps compared to clade D algae. While clade D *Symbiodinium* algae spread in polyps within 20 days of inoculation, clade C1 algae did not increase for nearly 2 months (Figs. 1 and 2). In addition, we conducted the same experiment using aposymbiotic polyps 2 months after metamorphosis. As a result, clade C1 hardly increased in polyps for two months while clade D spread in polyps in about two weeks. The rates of increase of clades C1 and D algae were identical, regardless of the time after metamorphosis (Figure S2). We consider two possibilities to explain this phenomena. First of all, is that corals suppress the number of symbiont by discharging or digesting clade C *Synbiodinium* and secondly is that only a small number of clade C might invade and stay inside the coral body during initial inoculation. As shown in Fig. 3, a large number of clade C *Synbiodinium* remained around polyps although we are uncertain whether these symbionts were discharged or not. In the field, clade C algae are not dominant in Acroporid juveniles [14], [8]–[9], suggesting that corals in the field may control the uptake of clade C algae during early growth stages. Nonetheless, Little et al. (2004) and Littman et al. (2010) showed that Acroporid juvenile polyps acquired clade C algae in their early life history [10]–[11]. Hence, the selectivity of *Symbiodinium* acquisition by juvenile polyps may depend on the coral's condition and surrounding environment. Some studies have investigated *Symbiodinium* selection mechanisms, and have found them to be mediated by lectin/glican interactions, which act as mechanisms of recognition in many pathogenic and mutualistic associations [24]–[25]. Wood-Charlson et al. (2006) reported that the blocking of symbiont cell-surface glycans with specific lectins resulted in decreased infection success of *Fungia scutaria* larvae [25]. Such lectin/glican interactions may be of concern with the different increased rate of clade C and clade D inside juvenile polyps. Furthermore, cell cycle of symbiotic dinoflagellate can be actively regulated by nutritional status [26]. There is a possibility that the difference in the increase rate of clade C and clade D cells inside of hosts is influenced by nutritional status.

### Differences between clade C and clade D symbiosis

We found that the green fluorescence of juvenile polyps was different depending on associations with different *Symbiodinium* clades (Fig. 3). When C1 *Symbiodinium* algae were introduced to juvenile polyps, a bright fluorescence was observed in the early stage of symbiosis. Since such a bright fluorescence was not observed in aposymbiotic corals and corals associated with clade D *Symbiodinium* algae, this was probably caused by an association with clade C1 algae. Fluorescence proteins exhibit significant hydrogen peroxide scavenging activities [27]. In corals, genes coding for fluorescent proteins change their expression under temperature stress [28], [25]. Heat stress could be attributed to greener of juvenile polyps [29], [16]. Bright fluorescence was observed in corals containing clade C1 algae, suggesting that corals experience oxidative stress when they initially acquire clade C1 *Symbiodinium* algae. Furthermore, to investigate the correlation of oxidative stress and fluorescence in corals, their antioxidant activity (catalase) was measured. Catalase is responsible for deactivating the reactive oxygen species, H_2_O_2_, into water and oxygen. It has been reported that catalase activities increase rapidly in coral tissues exposed to high temperature [19], [30]. In this study, catalase activity tended to be higher in corals with clade D algal symbiosis than in those with clade C1 algal symbiosis. Furthermore, 5-month-polyps had a higher level of catalase activity than 1-month-polyps, indicating that catalase activity may be influenced by increase in the number of endosymbiotic algae. Our results suggested that the bright fluorescence of polyps was not caused by oxidative stress from endosymbiotic algae. Another possible function of the green fluorescent protein is to be a key component in the immune system of corals. D'Angelo et al. (2012) demonstrated that melanin synthesis pathway activity (potential immune response) was correlated with increases in fluorescence [31]. Differences in fluorescence emission of polyps may have resulted from coral immune responses to each *Symbiodinium* algae.

In this study, corals associated with clade D algae grew faster than polyps associated with clade C1 algae under laboratory conditions. These results suggested that clade clade D algae contributed to skeletal growth during early growth stages, in comparison to clade C1. However, our results contradict others in the literature. For example, Little et al. (2004) reported that corals with clade C1 algae grew faster than those with clade D algae when the number of polyps was compared after 6 months [10]. Furthermore, Jones and Berkelmans (2013) compared the growth rate of *A. millepora* colonies with clades C2 and D algae by measuring their buoyant weight, and found that corals with clade D symbionts grew slower than those with clade C2 symbionts [14]. There are many questions regarding the different effects of clades C and D *Symbiodinium* algae on corals. In this study, one type of antioxidant activity (catalase activity) of corals was not significantly different between polyps associated with clade C1 algae and those associated with clade D algae. Therefore, oxidant stress from endosymbiotic algae likely does not affect the growth rate of juvenile polyps under non-stress conditions; clade differences in photosynthetic products and environmental responses may have influenced the growth rate of corals.

In conclusion, we succeeded in growing corals associated with monoclonal *Symbiodinium* algae in clades C1 and D, and demonstrated that clade differences can affect the growth rates and fluorescence of juvenile polyps. Clade D algae may contribute to the growth of juvenile corals and clade D algae may be more suitable for symbiosis in early growth stages. Furthermore, we suggest that these differences cannot be explained by differences in levels of oxidative stress. To understand the differences between clade C1 and clade D algal associations, further molecular studies are needed. However, our results might not be applied to field corals, because our used symbiotic polyps were cultivated in laboratory conditions. As a model symbiosis system, the corals polyps used in this study were useful to facilitate the molecular analysis. Since it is difficult to maintain juvenile polyps in the laboratory for extended periods, the number of polyps that can be used in experiments was restricted. The development of an incubation system that can maintain a large number of juvenile polyps for experiments, such as gene expression profiling, will be valuable in the future studies.

## Supporting Information

Figure S1 **RFLP pattern of cultured** ***Symbiodinium*** **algae and algae associated with corals.** Lane 1: clade C1 *Symbiodinium* culture. Lane 2: clade D *Symbiodinium* culture. Lane 3: Polyps 1 month after inoculation with clade C1 *Symbiodinium* algae. Lane 4: Polyps 1 month after inoculation with clade D *Symbiodinium* algae. Lane 5: Polyps 4 months after inoculation with clade C1 *Symbiodinium* algae. Lane 6: Polyps 4 months after inoculation with clade C1 *Symbiodinium* algae.
(TIF)

Figure S2 **Juvenile polyps of** ***Acropora tenuis*** **colonized by** ***Symbiodinium*** **monoclonal cells: CCMP2466 (clade C1) or CCMP2556 (clade D).** *Symbiodinium* algal cultures were introduced to aposymbiotic polyps 2 months after metamorphosis. Polyps were photographed 16, 33, 47, and 66 days after inoculation.
(TIF)
