# Supplementary figures and images for: Comparing the Effects of Symbiotic Algae (Symbiodinium) Clades C1 and D on Early Growth Stages of Acropora tenuis
Source: PLoS One. 2014 Jun 10;9(6):e98999. doi: 10.1371/journal.pone.0098999 (PMC4051649; doi:10.1371/journal.pone.0098999)

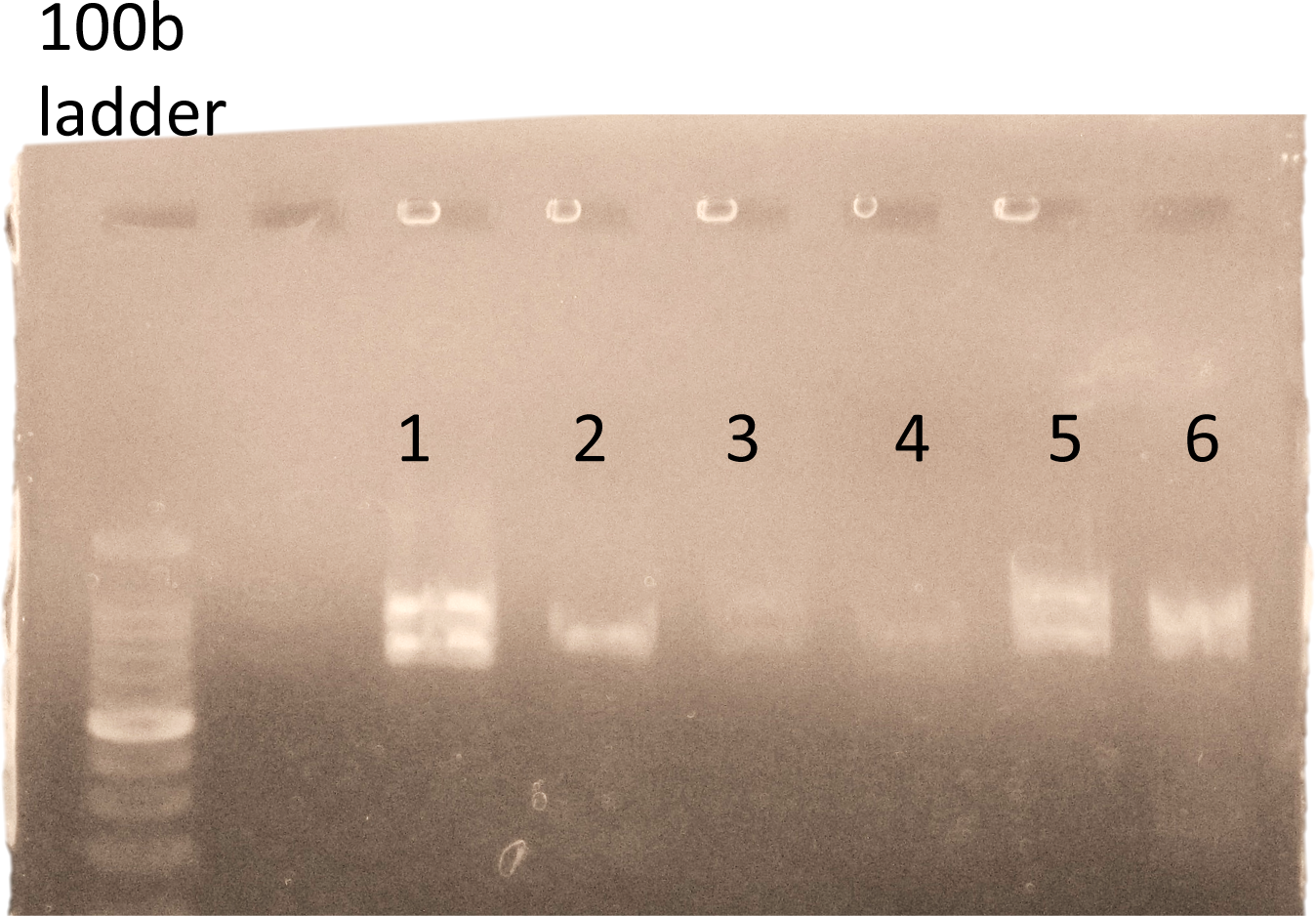

Supplement: Figure S1 — RFLP pattern of cultured Symbiodinium algae and algae associated with corals. Lane 1: clade C1 Symbiodinium culture. Lane 2: clade D Symbiodinium culture. Lane 3: Polyps 1 month after inoculation with clade C1 Symbiodinium algae. Lane 4: Polyps 1 month after inoculation with clade D Symbiodinium algae. Lane 5: Polyps 4 months after inoculation with clade C1 Symbiodinium algae. Lane 6: Polyps 4 months after inoculation with clade C1 Symbiodinium algae. (TIF) [file pone.0098999.s001.tif]

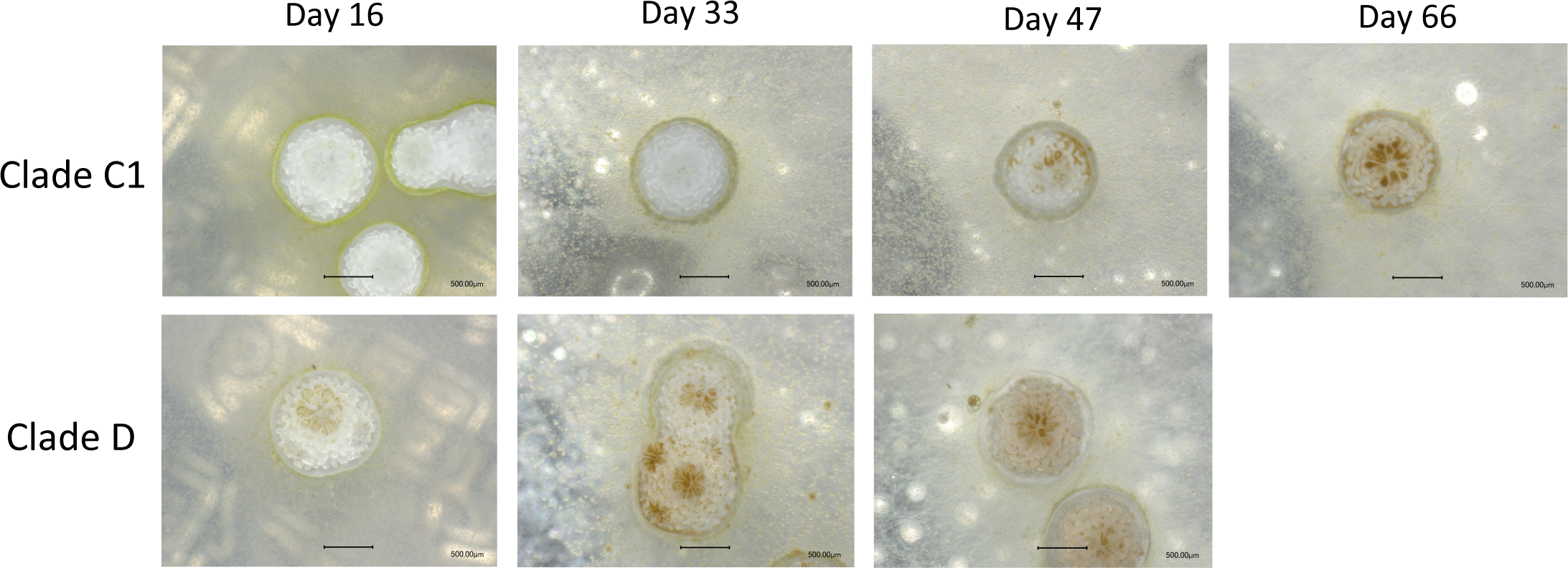

Supplement: Figure S2 — Juvenile polyps of Acropora tenuis colonized by Symbiodinium monoclonal cells: CCMP2466 (clade C1) or CCMP2556 (clade D). Symbiodinium algal cultures were introduced to aposymbiotic polyps 2 months after metamorphosis. Polyps were photographed 16, 33, 47, and 66 days after inoculation. (TIF) [file pone.0098999.s002.tif]
